# Arachidonic Acid Derived Lipid Mediators Influence Kaposi’s Sarcoma-Associated Herpesvirus Infection and Pathogenesis

**DOI:** 10.3389/fmicb.2019.00358

**Published:** 2019-03-12

**Authors:** Jayashree A. Chandrasekharan, Neelam Sharma-Walia

**Affiliations:** Department of Microbiology and Immunology, H.M. Bligh Cancer Research Laboratories, Chicago Medical School, Rosalind Franklin University of Medicine and Science, North Chicago, IL, United States

**Keywords:** KSHV, latency, cyclooxegenase-2, lipoxygenase, lipoxin A4

## Abstract

Kaposi’s sarcoma-associated herpesvirus (KSHV) infection, particularly latent infection is often associated with inflammation. The arachidonic acid pathway, the home of several inflammation and resolution associated lipid mediators, is widely altered upon viral infections. Several *in vitro* studies show that these lipid mediators help in the progression of viral pathogenesis. This review summarizes the findings related to human herpesvirus KSHV infection and arachidonic acid pathway metabolites. KSHV infection has been shown to promote inflammation by upregulating cyclooxygenase-2 (COX-2), 5 lipoxygenase (5LO), and their respective metabolites prostaglandin E_2_ (PGE_2_) and leukotriene B4 (LTB_4_) to promote latency and an inflammatory microenvironment. Interestingly, the anti-inflammatory lipid mediator lipoxin is downregulated during KSHV infection to facilitate infected cell survival. These studies aid in understanding the role of arachidonic acid pathway metabolites in the progression of viral infection, the host inflammatory response, and pathogenesis. With limited therapeutic options to treat KSHV infection, use of inhibitors to these inflammatory metabolites and their synthetic pathways or supplementing anti-inflammatory lipid mediators could be an effective alternative therapeutic.

## Introduction

Kaposi’s sarcoma-associated herpesvirus or human herpesvirus 8 (HHV8) is the causative agent of KS, PEL, MCD, and KICS (Cesarman and Knowles, 1999; Greene et al., 2007; Kalt et al., 2009; Ganem, 2010). KS lesions are characterized by proliferating spindle shaped endothelial cells while PEL is characterized by a null lymphocyte immunophenotype (CD45+, but neither T cells nor B cells) arising in body cavities (Chen et al., 2007; Saini et al., 2013). KS occurs in less than 1% of the population in the United States mostly found in HIV infected patients, while PEL, an aggressive AIDS-linked KSHV-associated non-Hodgkin’s lymphoma (NHL) occurs in about 4% of the population in the United States (Chen et al., 2007; Saini et al., 2013). While the number of AIDS-KS and PEL cases has been dramatically reduced in the United States since the widespread use of combined antiretroviral therapy, KSHV still causes major mortality in the developing world (Mesri et al., 2010). PEL has complications such as extracavitary KSHV associated solid lymphoma (solid PEL) without serous effusions that involves mainly extranodal tissues (De Paoli and Carbone, 2016). KSHV is detected in all clinical forms of KS, including classical KS, endemic KS in Africa, epidemic AIDS-related KS, and iatrogenic/organ-transplant KS. KICS is a newly characterized condition caused by lytic KSHV infection (Uldrick et al., 2010; Tamburro et al., 2012).

**FIGURE 1:**
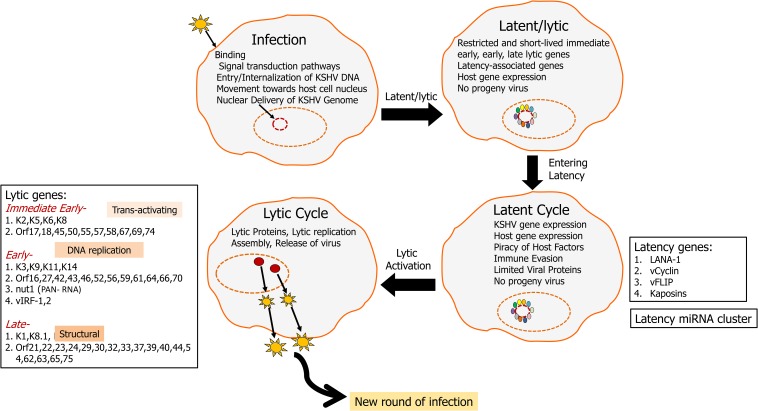
KSHV life cycle: KSHV infects a variety of cells including endothelial, epilthelial, B cells, monocytes, and keratinocytes. Upon infection, KSHV aims at lifelong persistence by adopting two lifestyles including a long persistent latency cycle and a short reproductive lytic cycle. The KSHV genome (165–170 kbp) consists of dsDNA that is linear within the viral particle but circularizes after it traverses to the host nucleus. This circular form is known as the viral episome (Hu et al., 2002; Si et al., 2008). During latency, the virus replicates via the host DNA synthetic machinery, viral genome remains as an episome tethered to the host chromosome and is characterized by the expression of a limited repertoire of genes, including LANA1, vCyclin, vFLIP, and kaposin. LANA1 is a predominant latency gene that plays an important role in episome maintenance and it drives latency. KSHV can then enter lytic cycle, which is marked by the viral replication, virion assembly, and release of infectious virions from the infected cells. Lytic genes can be classified into immediate early, early, and late. RTA (ORF50) is a critical lytic gene that helps the transition of genome from latency to the lytic cycle. During the lytic cycle, KSHV expresses many other proteins including K1 and viral interleukin 6 (vIL6), which plays important roles in promoting oncogenesis by activating multiple signaling pathways driving cellular growth.

Kaposi’s sarcoma-associated herpesvirus was discovered using representational difference analysis of DNA extracted from an AIDS-KS specimen and DNA from healthy skin of the same patient in 1994 by Yuan Chang, Patrick S. Moore, and colleagues (Chang et al., 1994; Boshoff et al., 1995a,b; Cesarman et al., 1995; Schulz and Weiss, 1995; Soulier et al., 1995). KSHV has a double-stranded DNA genome of approximately 165k bases inside a capsid, which is then surrounded by a proteinaceous layer called the tegument (Sathish et al., 2012). Upon infection, KSHV can undergo two distinct types of life cycle (Figure 1), lytic where the genome replicates to produce more virions and a latent cycle where the viral genome remains as an episome (Ye et al., 2011). Recently, with structured illumination microscopy (SIM), it was shown that KSHV tethers its genomes not only to nucleosome bound chromosomal DNA but also to nucleosome-bound viral DNA to form clusters of genomes that partition as units and that is predicted to help in the rapid establishment of high viral copy numbers in infected cells (Chiu et al., 2017). During both cycles, KSHV transcribes several genes which can subvert a variety of host cell processes such as cell death and proliferation (Murtadak et al., 2015), glucose uptake and energy management, immunosuppression, and inflammation (Mesri, 1999; Morini et al., 1999).

Latency is characterized by the expression of the following gene open reading frames *OrfK12/*Kaposin, viral FLICE, FADD-like interferon converting enzyme-like inhibitory protein (*Orf71/*K13/vFLIP), *Orf72/*vCyclin, latency associated nuclear antigen-1 (*Orf73/*LANA1), and a cluster made up of 12 pre-miRNAs (Gottwein, 2012; Qin et al., 2017). The KSHV lytic cycle is characterized by expression of over 80 genes classified into immediate early, early, and late, which aid in infectious virus production (Figure 1; Jenner et al., 2001). Immediate early genes such as *OrfK8* (bZIP), *Orf45*, and *OrfK4.2* help in a transition from latency to the lytic phase. Early lytic genes including PAN/nut-1/T1.1 RNA (*OrfK7*) and *OrfK14* encode for proteins involved in DNA replication while late lytic genes code for various structural proteins. Late lytic genes include *Orf22*, *Orf25*, and *Orf64* (Wakeman et al., 2017). *Orf50/*RTA (replication and transcriptional activator), master latency/lytic switch, an important player as both an initiator and a controller of KSHV lytic DNA replication is an immediate-early protein that disrupts the latency and promotes lytic cycle (Figure 1; Wakeman et al., 2017). *Orf50/*RTA auto-activates its own promoter and transactivates the expression of several downstream lytic genes such as *K5*, *K8*, *K2*, *K12*, *ORF6*, *ORF57*, *ORF74*, *K9*, *ORF59*, *vIL6*, *PAN-RNA*, *vIRF1*, *K1*, and *ORF65*, either through an RTA-responsive element (RRE) or via other viral regulatory genes (Deng et al., 2000).

**FIGURE 2:**
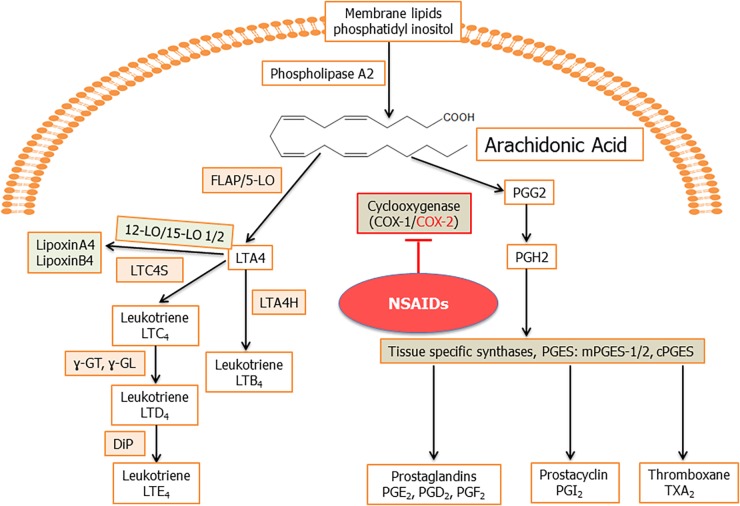
Schematic for arachidonic acid (AA) pathway: phospholipids from the plasma membrane are mobilized and converted to AA by phospholipase A2 (PLA_2_) enzyme activity. Cyclooxygenase 1 (constitutive) and cyclooxygenase 2 (inducible) enzymes catalyze the synthesis of prostaglandin H2 (PGH2) from AA through an unstable intermediate PGG2 (Vane et al., 1998; Smith et al., 2000). PGH2 is converted by microsomal prostaglandin E synthase (mPGES) to PGE_2_, which is released in the microenvironment. Besides PGE_2_, other prostanoids formed from PGH2 include PGI_2_, TXA_2_, PGD_2_, and PGF2α (Hyde and Missailidis, 2009). In human cells, generally, four types of LOs, namely, 5-, 12-, and 15-LO-1 and -2 have been identified. 5LO in conjunction with its activating protein, FLAP, catalyze the dioxygenation of AA into give 5-hydroperoxy eicosapentaenoic acid (5-HpETE). 5LO, with FLAP, then catalyze a second step, the dehydration of 5-HpETE to the intermediate LTA_4_, that leads to the formation of the leukotrienes and lipoxins (Hyde and Missailidis, 2009). Hydrolysis of LTA_4_ is catalyzed by LTA_4_ hydrolase (LTA4H) to produce LTB4. LTA_4_ is also metabolized by the enzyme LTC_4_ synthase to produce LTC_4_, which is acted upon by γ-glutamyl-transpeptidases to yield LTD_4_. LTD_4_ is acted upon by a dipeptidase (DiP) to produce LTE_4_. The combination of LO, -5, -12, or -15 on the same arachidonic acid molecule produces lipoxins.

Arachidonic acid is an unsaturated (ω-6 fatty acid) fatty acid which is transformed into a variety of lipid mediators to modulate primarily inflammatory reactions (Samuelsson, 1991). Arachidonic acid is released from the membrane by the action of phospholipase A2 (PLA2; Figure 2). Arachidonic acid is metabolized by the COX and LO pathways, to produce eicosanoids, which include PGs and LTs and their derivatives (Fanning and Boyce, 2013; Chandrasekharan et al., 2016; Figure 2). PLA2 also result in the formation of lysoglycero-phospholipids, which are the precursors for lysophosphatidic acid (LPA) and sphingosine-1-phosphate (S1P; Fanning and Boyce, 2013; Chandrasekharan et al., 2016). The arachidonic acid pathway widely influences the process of inflammation through its lipid mediators (Bennett and Gilroy, 2016). These lipid mediators are known to play a pivotal role(s) in cancer and inflammation associated diseases like arthritis, allergy, and asthma (Greene et al., 2011). Viral infection also alters the lipid mediators of this pathway to establish infection and pathogenesis (Chandrasekharan et al., 2016; Figure 2).

Kaposi’s sarcoma-associated herpesvirus is known to alter several host pathways for its survival and one such extensively studied is the arachidonic acid pathway (Sharma-Walia et al., 2006, 2010b, 2012, 2014; George Paul et al., 2010; Paul et al., 2011, 2013a,b; Chandrasekharan and Sharma-Walia, 2015; Marginean and Sharma-Walia, 2015; Chandrasekharan et al., 2016). KSHV triggers a pro-inflammatory microenvironment to promote its latency and pathogenesis (Chandrasekharan et al., 2016). Arachidonic acid pathway is a key connection between KSHV infection and its associated inflammatory responses (Mesri, 1999; Zhong et al., 2017). Having stated the importance of the arachidonic acid pathway in KSHV infection/pathogenesis one can envisage the role of lipid mediators in the treatment of KS and PEL. This review summarizes the key findings of the role of several enzymes and metabolites of the arachidonic acid pathway during KSHV infection.

## Role of Arachidonic Acid Metabolism in KSHV Infection and Pathogenesis

### Cyclooxygenase-2, a Prominent Enzyme Upregulated During KSHV Infection

Cyclooxygenase, an enzyme involved in the synthesis of PGs, regulated by the popular non-steroidal anti-inflammatory drugs (NSAIDs; Sasaki et al., 2017). Aspirin inhibits the COX pathway by downregulating the levels of pro-inflammatory PGs, particularly PG E2 (PGE_2_; Vane and Botting, 1997; Botting, 2010). COX-1 and COX-2 are the two isoforms of this enzyme (Williams et al., 1999a,b,c; Rouzer and Marnett, 2009; Greene et al., 2011; Schneider et al., 2011; Yu et al., 2016; Grosch et al., 2017) with COX-1 produced constitutively while COX-2 is inducible (Dubois et al., 1998; Figure 2). COX-1 and COX-2 catalyze two sequential reactions to initiate the COX pathway and produce PGG2. An endoperoxidase then reduces PGG2 to PGH2. PGH2 then gets converted into bioactive prostanoids PGD_2_, PGE_2_, PGF_2α_, PGI_2_, and TXA_2_ by H-PGDS, mPGESs mPGES-1, mPGES-2/cytosolic PGES, PGF synthase (PGFS), PGI synthase (PGIS), and TXS, respectively (Fanning and Boyce, 2013; Chandrasekharan et al., 2016; Figure 2). Cycloxygenase is a clinically important enzyme and its inhibitors are widely used to treat inflammation (Fitzpatrick, 2004; Suleyman et al., 2007; Greene et al., 2011; Yu et al., 2016; Grosch et al., 2017; Figure 2).

*De novo* KSHV infection of primary HMVEC-d or HFF cells is a good *in vitro* model for KS and is characterized by the sustained expression of latency-associated *ORF73/*LANA1, *ORF72/*vCyclin, and *ORF71/*K13/vFLIP genes (Krishnan et al., 2004). However, a unique aspect of this *in vitro* infection is the concurrent transient expression of a limited number of lytic KSHV genes, such as the lytic cycle switch gene *ORF50/*RTA and the immediate early lytic *K5*, *K8*, and *vIRF2* genes (Krishnan et al., 2004). In the first step of identifying host molecules involved in KSHV pathogenesis, a variety of genes involved in cellular apoptosis, transcription, cell cycle, signaling, inflammatory response and angiogenesis were identified and COX-2 was one of the upregulated genes (Naranatt et al., 2004). Further studies performed on HMVEC-d cells infected with KSHV for various time points showed that COX-2 levels were induced as early as 30 min postinfection, reached a high level at 2 h and gradually started returning to basal level by 72 h (Sharma-Walia et al., 2006). No change in the level of COX-1 was observed in endothelial cells infected with KSHV (Sharma-Walia et al., 2006). COX-2 induction could be triggered by KSHV binding and entry while the augmented levels require KSHV genome (Sharma-Walia et al., 2006). This was identified by screening levels of COX-2 in HMVEC-d cells infected with UV inactivated KSHV. UV inactivated KSHV was generated by inactivating KSHV in UV (365 nm) for 20 min. UV inactivated KSHV efficiently binds and enters into host cells but it does not express viral genes (Sharma-Walia et al., 2004). UV inactivated KSHV infection could enhance COX-2 levels suggesting the role of KSHV binding and entry stages of infection involving the interplay of viral glycoprotein’s (Sharma-Walia et al., 2006). Like COX-2, the level of its metabolite PGE_2_ was elevated at 2 h post infection and gradually decreased to basal levels at 72 h (Sharma-Walia et al., 2006). Since COX-2 induction by UV-inactivated KSHV suggested stimulation during the binding and entry stages of infection, the ability of KSHV envelope glycoproteins gB and gpK8.1A to induce COX-2 was examined (Sharma-Walia et al., 2006). Both glycoproteins induced COX-2 but to a lesser extent than KSHV live virus, suggesting that viral gene expression early during infection, and possibly together with viral gene-induced host genes are probably essential for the increased and sustained induction of COX-2 and PGE_2_ (Sharma-Walia et al., 2006).

## Outcomes of Elevated COX-2

Since elevated levels of COX-2 were found in KS patient tissue sections, its role in pathogenesis events such as secretion of inflammatory cytokines, angiogenesis, cell survival, and invasion were explored (Sharma-Walia et al., 2010b). HMVEC-d cells infected with KSHV at various time points secreted a high level of inflammatory cytokines such as growth regulated oncogene (GRO), GROα, IL1α, IL1β, ILs-(2, 3, 6, 7, and 12-p40), TNFα, TNFβ, and SDF-1 [a ligand for the chemokine receptor CXCR4 or fusin or CD184 (cluster of differentiation 184)], and IFNγ in their spent culture supernatants (Sharma-Walia et al., 2010b). The levels of these inflammatory cytokines were constantly increased 2 h post infection and at 8 h post infection reached a high (3–3.5-fold increase) level. Chemokines such as RANTES (cytokine regulating T cell response), MCPs-2 and 3, thymus and activation-regulated chemokine, MIP, macrophage derived chemokine, monokine induced by IFN-γ, epithelial neutrophil-activating peptide and inflammatory cytokine were found upregulated in *de novo* KSHV infected HMVEC-d cells. Similarly, several growth and angiogenic factors such as EGF, insulin-like growth factor-1, platelet-derived growth factor-BB (PDGF-BB), macrophage colony stimulating factor, G-CSF, GM-CSF, angiogenin, oncostatin-M, thrombopoietin, VEGF, stromal cell derived factor-1, stem cell factor, TGFβ1, and leptin were elevated in KSHV infected HMVEC-d cells.

To validate the involvement of KSHV induced COX-2 on inflammatory cytokines and angiogenic factors, HMVEC-d cells were either pretreated with COX-2 inhibitors NS-398 or indomethacin or silenced for COX-2 gene expression (Sharma-Walia et al., 2010b). VEGF-A, VEGF-C (angiogenic molecules), GRO (cytokine with inflammatory and growth-regulatory properties), RANTES, and SDF-1 were alleviated upon blocking COX-2 (Sharma-Walia et al., 2010b). IL-8 was not affected by COX-2 inhibition (Sharma-Walia et al., 2010b). Many of the observed upregulated molecules such as PGE_2_, VEGF, and b-FGF play a vital role in endothelial cell migration and tube formation. KSHV-induced COX-2 enhanced the levels of these molecules and promoted endothelial cell tube formation, while this effect was reversed upon blocking COX-2 using chemical inhibitors (Sharma-Walia et al., 2010b). VEGF-A levels were reduced in latently infected TIVE-LTC cells treated with COX inhibitors, plus a significant reduction in capillary tube formation was observed (Sharma-Walia et al., 2010b). MMPs play an important role in maintaining tissue homeostasis and are often seen to be upregulated in several cancers. KSHV infection enhanced expression and secretion of MMPs including MMP-1, -2, -8, -9, and -13 (Sharma-Walia et al., 2010b). KSHV-induced COX-2 significantly induced MMP-2 and -9 in *de novo* infected HMVEC-d cells and blocking it drastically reduced their levels (Sharma-Walia et al., 2010b). This study demonstrated that COX-2 and its metabolite PGE_2_ are upregulated during KSHV infection of primary endothelial cells; KSHV utilizes those to target several pathways that facilitate pathogenesis and angiogenesis (Sharma-Walia et al., 2010b).

## Understanding COX-2 Regulation by KSHV Infection

Since COX-2 and PGE_2_ are upregulated during KSHV infection, understanding the mechanism of induction could help in designing therapeutic strategies for KS and PEL. To determine if regulation occurs at the transcriptional level, HMEVC-d cells were pretreated with transcriptional inhibitor actinomycin D prior to *de novo* KSHV infection (Sharma-Walia et al., 2010b). Pretreatment of HMVEC-d cells with actinomycin D drastically inhibited the effect of KSHV infection on both COX-2 mRNA levels and PGE_2_ secretion. To determine if a KSHV-induced signal cascade and/or viral gene expression is required for COX-2 induction, COX-2 promoter activity in HMVEC-d cells were monitored. These HMVEC-d cells were transfected with the COX-2 promoter and then infected with 30 DNA copies of KSHV/cell (either live or UV inactivated and replication incompetent; Sharma-Walia et al., 2010b). Compared to live virus UV-KSHV could not induce sustained levels of COX-2, thus suggesting that virus entry and binding induces COX-2 but KSHV gene transcription is required to sustain COX-2 (Sharma-Walia et al., 2010a). It is well known that COX-2 promoter activity is regulated by several transcription factors, including NFkB, nuclear factor IL-6, activator protein AP-1, cAMP response element binding (CREB) protein, and nuclear factor of activated T cells (NFAT; Iniguez et al., 1999, 2000). The COX-2 promoter has distal NFAT (dNFAT) and proximal NFAT (pNFAT) *cis*-acting elements. Promoter assays show that dNFAT plays an important role in KSHV-induced COX-2 promoter activity (Sharma-Walia et al., 2010a). cAMP response element (CRE) recognition sequences in the human COX-2 promoter are also important for KSHV infection-induced transcriptional activity as promoter deletion experiments show that these regions are vital for transcriptional activation upon KSHV infection. CRE and NFAT together are important for KSHV infection-induced COX-2 regulation (Sharma-Walia et al., 2010b). To identify the role of KSHV-induced signal transduction pathways in COX-2 regulation, several signaling inhibitors were used to study COX-2 promoter activity and its gene expression. PI3K, PKC, FAK, MEK, P38, and JNK inhibition strongly reduced COX-2 promoter activity and its gene expression (Sharma-Walia et al., 2010a). PGE_2_ being the end product its influence on COX-2 promoter and gene expression was studied by supplementing PGE_2_ exogenously (Sharma-Walia et al., 2010a). It was found that PGE_2_ could stimulate both COX-2 promoter activity and gene expression concluding that PGE_2_ autoregulates COX-2 (Sharma-Walia et al., 2010a). PGE_2_ amplifies COX-2 levels by cAMP/PKA signaling mediated inactivation of GSK3 (Sharma-Walia et al., 2010a).

In another study, the interplay between KSHV latency protein vFLIP and host protein COX-2 was demonstrated (Sharma-Walia et al., 2012; Paul et al., 2013b). Studies showed that vFLIP activates COX-2/PGE_2_ in a NF-κB-dependent manner and conversely, COX-2/PGE_2_ is required for vFLIP induced NF-κB activation, extracellular matrix (ECM) interaction, FAK/Src/AKT, Rac1-GTPase activation, mitochondrial antioxidant enzyme manganese superoxide dismutase (MnSOD) level, and anoikis resistance (Sharma-Walia et al., 2012; Paul et al., 2013b). vFLIP expression mediated the secretion of pro-inflammatory cytokines, and spindle cell differentiation activated the phosphorylation of p38, RSK, FAK, Src, Akt, and Rac1-GTPase (Sharma-Walia et al., 2012; Paul et al., 2013b). Celecoxib, a selective inhibitor of COX-2, has demonstrated its chemotherapeutic properties in a variety of cancers including colon, breast, skin, prostate, and pancreatic cancer cells, but was never tested in KSHV-associated malignancies (Jendrossek, 2013). Blocking COX-2 with celecoxib or NS398 [*N*-(2-cyclohexyloxy-4-nitrophenyl)-methanesulfonamide] decreased activation of signaling molecules including NFKB (reduced nuclear translocation of p65), FAK, Src, AKT, ERK, P-38, RSK, and Rac1-GTPase, and thereby decreased vFLIP-mediated protection of HMVECs from anoikis (Sharma-Walia et al., 2012). KSHV vGPCR (G-protein coupled receptor), vFLIP have been shown to induce expression of COX-2/PGE_2_ but not COX-1, and its paracrine effects in KS pathogenesis (Matta et al., 2007; Shelby et al., 2007). Another elegant study linked STE20 kinase family member-MAP4K4 (MEK kinase kinase kinase) to COX-2, which contributed to KSHV lytic reactivation (Haas et al., 2013; Mariggio et al., 2017).

## Utilizing COX-2 as a Therapeutic Target

To determine the role of KSHV induced COX-2 on the secretion of PGE_2_, indomethacin and NS 398 (inhibitors of COX-2) were used to pretreat KSHV infected HMVEC-d cells. A 93 and 88% reduction in levels of PGE_2_ was observed thereby concluding that KSHV infection associated COX-2 upregulation was the reason for enhanced PGE_2_ levels. Pretreating HMVEC-d and HFF cells with COX-2 inhibitors did not interfere in KSHV binding, entry, and trafficking (Paul et al., 2011). However, viral latency gene *Orf73*/LANA1 was significantly reduced by 80% at 24 h post infection in HMVEC-d cells pretreated with indomethacin, an NSAID (Ghosh et al., 2015). The level of lytic gene *Orf50/*RTA was not affected by indomethacin or NS-398 pretreatment. NS-398 is a COX-2-specific inhibitor that has been shown to have chemotherapeutic potential against colon and pancreatic cancer cells (Paul et al., 2011). Exogenous PGE_2_ supplementation could reverse this inhibitory effect of indomethacin and NS-398 (Sharma-Walia et al., 2006). Treating PEL cells with orally active COX-2 selective inhibitor Nimesulide (4-nitro-2-phenoxymethanesulfonanilide) to disrupt latency has shown to be beneficial (Paul et al., 2011). Effect of nimesulide treatment was tested on PEL (BCBL-1, BC-3, JSC-1), EBV-infected (Raji) and non-infected (Akata, Loukes, Ramos, BJAB) human Burkitt’s lymphoma (BL), and EBV harboring lymphoblastoid (LCL), KSHV infected (TIVE-LTC), TIVE, and primary endothelial HMVEC-d cells (Paul et al., 2011). KSHV positive PEL cells (BCBL-1, BC-3) reduced cell proliferation, colony formation, and enhanced apoptosis when compared to KSHV negative B-lymphoma cells (BJAB; Paul et al., 2011). Nimesulide treatment of PEL cells blocked LANA1 and vFLIP latency genes. Targeting COX-2 is effective in downregulating latency in PEL cells; however, further studies are required using *in vivo* models as these inhibitors are associated with cardiovascular diseases (Pawlosky, 2013).

## Role of Prostaglandin E_2_ (E-Type Prostanoid Receptors, EP) Receptors in KSHV Infection

**FIGURE 3:**
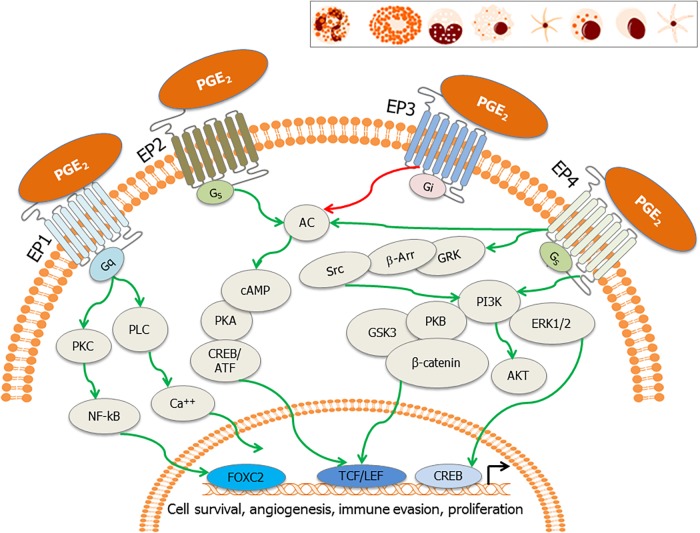
EP receptors and their downstream signaling: PGE_2_ binds to its cognate receptors, the E-series of prostaglandin receptors (EP1–EP4) on the plasma membrane and nuclear membrane. These receptors are also present on tumor cells, stromal cells and immune effector cells such as neutrophils, mast cells, monocytes, macrophages, myeloid dendritic cells, natural killer (NK) cells, plasma cells, and lymphoid dendritic cells (inset). Major signaling pathways involve activation of activation of phospholipase C (PLC) resulting in the increase in intracellular calcium (EP1), also the activation of PKC, NF-κB, adenylate cyclase; AC (EP2 and EP4) and subsequent surge in cAMP, and inhibition of adenylate cyclase (EP3). EP4 stimulates cAMP-independent signaling through activation of GRK/β-arrestin/Src/PI3K/GSK3 pathway, leading to the nuclear translocation of β-catenin. These signaling pathways subsequently induce the nuclear translocation/activation of transcription factors, which bind to the promoters of genes involved in cell cycle survival, anti-apoptosis, angiogenesis, immune evasion, and cell proliferation.

Prostaglandin E_2_, the most versatile prostanoid exerts its pleiotropic effects through a group of four G-protein-coupled receptors (GPCRs) designated subtypes EP1, EP2, EP3, and EP4 (Sugimoto and Narumiya, 2007; Yokoyama et al., 2014; Figure 3). These GPCRs have seven transmembrane helices (Funk, 2001). There are multiple splicing isoforms of the subtype EP3 (α, β, γ), which are generated by alternative splicing of the C-terminal tail (Sugimoto and Narumiya, 2007). Among all EP receptors, EP1 is known to regulate Ca2+ channel gating (Sugimoto and Narumiya, 2007) and increase intracellular calcium via receptor-activated Ca2+ channels (RACC; Katoh et al., 1995; Tabata et al., 2002; Figure 3). EPs, both EP2 and EP4 are coupled to induction of cAMP via adenylate cyclase (AC; Sugimoto and Narumiya, 2007; Figure 3). However, EP3 receptor inhibits AC and decreases cAMP concentrations (Sugimoto and Narumiya, 2007; Figure 3). EP4 is widely distributed in the body and it can activate PI3K and can be coupled to GPCR kinases (GRK), β-arrestin, and β-catenin signaling pathways during carcinogenesis (Yokoyama et al., 2013). Therapeutic potential of EP modulators has been shown in colorectal cancer, neurologic disease, glaucoma, ocular hypertension, bone formation, cardiovascular disease, ulcerative colitis, solid tumors, and B cell lymphoma (Markovic et al., 2017). Functional EP receptors EP1, EP2, EP3α, and EP4 have been reported to be present on perinuclear sites and nuclear membranes of a variety of cell types and tissues but the signaling pathways for these have not been investigated (Gobeil et al., 2003, 2006; Zhu et al., 2006).

Cyclooxygenase-2 has been shown as a major player of tumor progression in melanoma as COX-2 has strong relationship with the dynamic expression of programmed cell death protein ligand 1 (PD-L1) on tumor cells (Botti et al., 2017). The tumor-induced PGE_2_ due to overexpression of PGE2 synthase 1 and reduction of PGE_2_ degrading enzyme 15-hydroxyprostaglandin dehydrogenase induces PD-L1 expression in bone marrow-derived cells, macrophages, and myeloid-derived suppressor cells (MDSCs; Prima et al., 2017). COX2 inhibitors or PGE_2_ receptors EP2 and EP4 antagonists combined with anti-PD-1 blockade have shown the therapeutic potential in improving eradication of tumors and augmenting the numbers of functional tumor-specific cytotoxic T lymphocytes (CTL) function in tumor-bearing hosts (Miao et al., 2017). Follicular dendritic cells (FDCs) of the lymphoid tissue produce PGE_2_, and it plays an immunoregulatory function. Targeting PGE_2_ receptors using EP2 and EP4 antagonists along with COX-2 inhibitors results in synergistic inhibition of PGE_2_-mediated Akt phosphorylation/activation in FDC like (Harizi et al., 2003; Lee et al., 2008).

Prostaglandin E_2_ suppresses effector functions of macrophages and neutrophils and the Th1-, CTL-, and NK cell-mediated immunity (Kalinski, 2012; Agard et al., 2013; Turcotte et al., 2017). PGE_2_ promotes Th2, Th17, and regulatory T cell responses, impairs CD4+ T cell activation (Chemnitz et al., 2006; Kalinski, 2012; Sreeramkumar et al., 2012), and also tempers chemokine production (Kalinski, 2012). PGE_2_ can promote the tissue influx of neutrophils (Yu and Chadee, 1998), macrophages (Nakayama et al., 2006), and mast cells (Weller et al., 2007). PGE_2_ suppresses the cytolytic effector functions of NK cells (Bankhurst, 1982; Goto et al., 1983). PGE_2_ inhibits NK cell production of IFN-γ, abolishes NK cell “helper” function in the DC-mediated induction of Th1 and CTL responses (Mailliard et al., 2005), and facilitates the establishment of metastases in experimental animals (Yakar et al., 2003). PGE_2_ inhibits granulocyte functions (Smith, 1977) and underwrites to the defective innate host defense in patients after bone marrow transplantation (Ballinger et al., 2006). PGE_2_ limits the phagocytosis by alveolar macrophages and their pathogen-killing function (Serezani et al., 2007) partly via EP2 (Aronoff et al., 2004). PGE_2_ promotes mast cell induction, their local attraction, and degranulation via EP1 and EP3 (Hu et al., 1995; Gomi et al., 2000; Nakayama et al., 2006; Wang and Lau, 2006). Murine splenic NK cells express all EP1-4 receptors and inhibiting PGE_2_ production or preventing signaling through the EP4 receptor could suppress NK cell functions including migration, cytotoxicity, and cytokine release (Holt et al., 2012; Park et al., 2018).

Prostaglandin E_2_ disturbs early stages of DC differentiation and adds to systemic DC dysfunction in cancer (Kalinski et al., 1997; Sombroek et al., 2002; Sharma et al., 2003; Heusinkveld et al., 2011). PGE_2_ subdues the differentiation of functionally competent Th1-inducing DCs and the subsequent “PGE_2_ DCs” characterize MDSCs, which can suppress CTL responses (Obermajer et al., 2011). Besides these functions, PGE2 acts as an inhibitory damage-associated molecular pattern (DAMP) and negatively regulates cell death-induced inflammatory responses, which may have translational magnitudes in therapeutic interventions for inflammation-associated diseases (Hangai et al., 2016).

**FIGURE 4:**
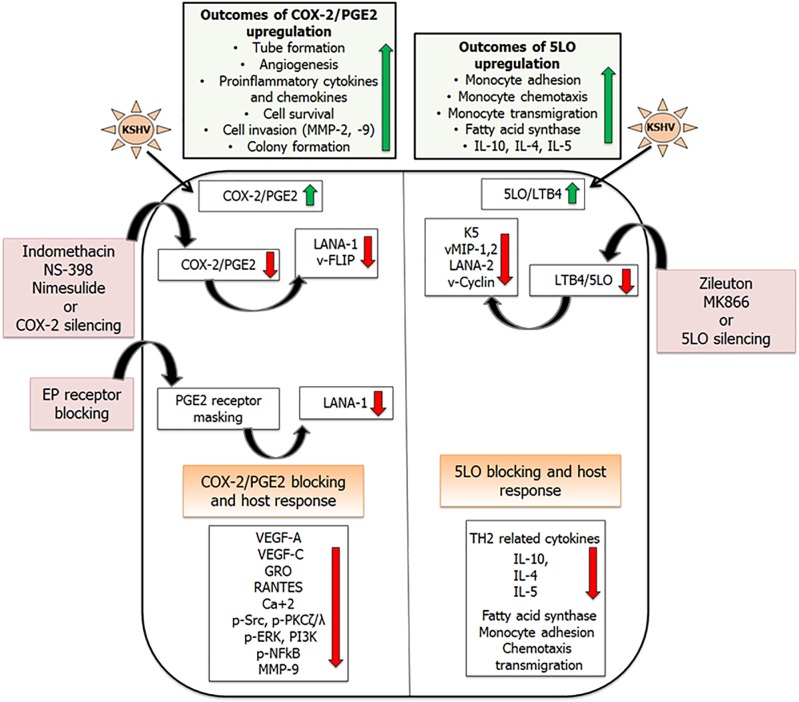
Combating inflammation promoted by KSHV infection: KSHV infection promotes pro-inflammatory lipid mediators of arachidonic acid pathway and the enzymes involved in their synthesis. Inhibiting these lipid mediators and the enzymes involved in their synthesis has shown efficacy in downregulating KSHV latent gene expression. This schematic has been divided into two sections showing inflammatory COX-2 (Sharma-Walia et al., 2006, 2010a,b, 2012; George Paul et al., 2010; Paul et al., 2011, 2013a,b), and LO pathways induced upon KSHV infection (Sharma-Walia et al., 2014). Upregulation of COX-2/PGE_2_ in KSHV infected cells promotes tube formation, angiogenesis, pro-inflammatory cytokine and chemokine release, cell survival, and cell invasion. 5LO/LTB_4_ upregulation promotes monocyte adhesion, chemotaxis, and transmigration. Reducing the levels of these lipid mediators by using their specific inhibitors (indomethacin, nimesulide, NS-398, Zileuton, MK866), gene silencing, or blocking PGE_2_ receptors (EP receptors) showed promise in downregulating KSHV gene expression (LANA1, vFLIP, vCyclin, LANA2, K5, vMIPs), angiogenesis (VEGFs), inflammatory cytokines (GRO, RANTES), and inflammation associated signaling cascades (pSrc, PI3K, and pNFκB; Sharma-Walia et al., 2006, 2010a,b, 2012, 2014; George Paul et al., 2010; Paul et al., 2011, 2013a,b).

Previous studies (Sharma-Walia et al., 2006, 2010a,b, 2012; George Paul et al., 2010; Paul et al., 2011, 2013a,b) revealed that COX-2 and its metabolite PGE_2_ are two pivotal molecules controlling KSHV latency. PGE_2_ binds to EP1-4 receptors (Buchanan and DuBois, 2006; Dubois, 2014). KS patient tissue section staining revealed higher levels of PGE_2_ receptors EP1-4 and mPGES when compared to tissue sections obtained from healthy controls (George Paul et al., 2010). TIVE-LTC exhibited upregulated expression of EP1, 3, and 4 with downregulated EP2. Blocking EP1 in TIVE-LTC cells reduced KSHV latency protein LANA1 while blocking EP4 downregulated COX-2 gene expression and PGE_2_ levels suggesting that KSHV utilizes EP receptors to maintain latency and COX-2/PGE_2_ levels. Blocking EP receptors also downregulated several cell signaling molecules such as pPI3K, pPKCζ/λ, Ca2+, pNFκB, and pERK, which impact KSHV infection and pathogenesis (Figure 4). EP1 (8-chloro-2-[3-[(2-furalphanylmethyl)thio]-1-oxopropyl] hydralphazide, dibenze [b,f] oxalphazepine-10 (11H)-calpharboxylic acid) and 2 (6-isopropoxy-9-oxoxanthene-2-carboxylic acid) antagonists reduced levels of pSrc. EP1, 2, and 3 antagonists reduced levels of p-PI3K. In addition, EP2 and EP4 (4-(4,9-diethoxy-1, 3-dihydro-1-oxo-2H-benz[f]isoindol-2-yl)-N-(phenylsulfonylbenzenacetamide)) antagonists downregulated p-PKCζ/λ whereas EP1, EP2, and EP4 antagonists treatment abrogated p-NFκB activation (George Paul et al., 2010). The mechanism of EP receptor regulation of LANA1 was found to be linked to its Ca2+ inducing property (George Paul et al., 2010). KSHV induced PGE_2_ induced Ca2+ levels, which sequentially enhanced signaling via EP1 receptor (George Paul et al., 2010). PGE_2_ then alters the LANA promoter in the −262 and −159 region. Blocking Ca2+ abrogated the effect of PGE_2_ induced LANA1 (George Paul et al., 2010). This study helped in understanding latency in a whole new breadth of calcium signaling via EP receptors in KSHV infected cells. This study advanced the role of COX-2 and PGE_2_ in latency establishment (George Paul et al., 2010). Studies were performed to understand how blockade of both COX and EP receptors would work in combination to treat KSHV infection and pathogenesis (Paul et al., 2013a). A combination of COX-2 inhibitor celecoxib, EP1 antagonist SC-51322, and EP4 antagonist GW 627368X induced apoptosis and promoted expression of tumor suppressors such as ATM, FHIT, HIC1, MCL1, NCAM1, RASSF1, TIMP2, TIMP3, and TP53 in PEL cells. One of the major issues with this treatment is the risk of thrombotic and cardiovascular events due to the absence of aspirin-like platelet aggregation inhibiting properties (Paul et al., 2013a). Due to the abundance of EP2 and EP4 receptors in cancer, it is considered as an appropriate target for treating breast and colorectal cancer (Reader et al., 2011; Ma et al., 2015). The highly specific and potent EP4 antagonist BGC20-1531 was also under clinical trial for treating headaches caused by a PGE_2_ outburst (Cai et al., 1999).

### 5-Lipoxygenase (5LO), an Enzyme of the Leukotriene Pathway, Upregulated During KSHV Infection

Yet another pro-inflammatory metabolite of the arachidonic acid pathway is leukotriene B4 (LTB_4_). Enzyme arachidonate 5-oxidoreductase [EC1.13.11.34] (5LO), its activating protein FLAP (5LO activating protein), and LTA4H are responsible for the synthesis of potent chemotactic LTB_4_ (Haeggstrom, 2004). LTB_4_ has been shown to impact the immune response and spread of viral infections, including those caused by HIV, respiratory syncytial virus (RSV), and Epstein-Barr virus (EBV). 5LO, LTA4H, and LTB_4_ are found at high levels in most of the inflammation and oxidative stress-associated cancers, such as breast, lung, prostate, pancreatic, colon, bladder, esophageal, and testicular cancers; glioma; chronic myelogenous leukemia; Mantle cell lymphoma (MCL); and NHLs. 5LO pathway inhibitors have been tested as chemoprevention agents in many cancers. Since LTB_4_ is linked to inflammation and immune modulation and KS, PEL, and MCD are chronic inflammation-associated malignancies, the regulation of 5LO/LTB_4_ cascade was tested as one of the virus’s triggered host factors involved in KSHV pathogenesis. It was demonstrated that KSHV infection induces the 5LO/LTB_4_ cascade to support its latent infection and in the induction of the inflammatory milieu (Sharma-Walia et al., 2014). Targeting of 5LO/LTB_4_ provides a new avenue of treatment against KSHV associated malignancies (Sharma-Walia et al., 2014).

To study the role of 5LO and LTA4H in KS, the skin tissue sections of healthy subjects and KS patients for the presence of 5LO and LTA4H were analyzed. KS patient tissue sections showed higher levels of 5LO and LTA4H when compared to healthy patient tissue sections (Sharma-Walia et al., 2014). 5LO is generally found in the cytoplasm but on activation it moves to the nuclear membrane with the help of FLAP to actively synthesize LTB_4_. 5LO also requires Ca2+ or NF-κB for its nuclear translocation. Latently infected TIVE-LTC cells showed a dense staining for 5LO in the nucleus when compared to uninfected TIVE cells (Sharma-Walia et al., 2014). The presence of 5LO in the nucleus determines its active state. This indicated that TIVE-LTC cells have a higher capacity to synthesize LTB_4_ when compared to TIVE cells (Sharma-Walia et al., 2014). TIVE-LTC cells also express high levels of LTA4H and FLAP enzymes when compared to TIVE cells (Sharma-Walia et al., 2014). HMVEC-d cells were *de novo* latently infected with KSHV and the status of 5LO pathway genes was examined. When compared to uninfected control HMVEC-d cells, latently infected HMVEC-d cells expressed higher levels of 5LO, LTA4H, and FLAP. Similar upregulation of 5LO pathway enzymes was seen in KSHV infected BCBL-1 than KSHV negative BJAB cells (Sharma-Walia et al., 2014). The induction of 5LO was dependent on the presence of the KSHV transcriptome, as UV-KSHV was unsuccessful in upregulating 5LO levels (Sharma-Walia et al., 2014). As KSHV infection has been shown to enhance levels of enzymes that synthesize LTB_4_, the level of this metabolite was measured in latently infected HMVEC-d cells. When compared to uninfected cells, latently infected HMVEC-d cells secreted high levels of LTB_4_. Cytoplasmic and nuclear fractions were isolated from KSHV infected HMVEC-d cells and TIVE LTC cells then the levels of 5LO, FLAP, and LTA4H were evaluated against respective controls. When compared to uninfected HMVEC-d cells, KSHV infected HMVEC-d cells showed high levels of 5LO, FLAP, and LTA4H in the nuclear fractions. A similar enrichment of 5LO pathway enzymes was seen in the nuclear fractions of TIVE-LTC when compared to TIVE cells (Sharma-Walia et al., 2014).

## Inhibiting 5LO Pathway

5 Lipoxygenase is localized in the nucleus as well as the cytosol, but on cellular activation, 5LO undergoes Ca+2 or NF-κB-dependent translocation to the nuclear envelope (Balcarek et al., 1988; Dixon et al., 1988; Funk, 2001; Peters-Golden and Brock, 2001). The mechanism of nuclear translocation involves the necessary association of 5LO with a novel 18-kDa membrane protein known as 5LO-activating protein, or FLAP (Miller et al., 1990). KSHV infection has already been shown to upregulate NF-κB (Cannon et al., 2003; Sadagopan et al., 2007). Treating BCBL-1 cells with NF-κB inhibitor Bay11-7082 has shown to drastically reduce the levels of 5LO (Figure 4). 5LO activation also requires Ca2+ for its nuclear translocation. Using Ca2+ inhibitors BAPTA-AM and TMB-8 showed no change in levels of 5LO suggesting that KSHV-induced NF-κB plays an important role in 5LO activation (Sharma-Walia et al., 2014). KSHV infection promoted 5LO and FLAP interaction (Sharma-Walia et al., 2014). Blocking 5LO either by silencing (5LO short hairpin RNA (h) lentiviral particle (5LO)-transduction) or by using inhibitors (MK866 and zileuton) reduced the levels of LTB_4_ secreted (Sharma-Walia et al., 2014). MK866 is an orally active anticancer drug that blocks binding of 5LO to the membrane by specifically interacting with the membrane-bound activating protein FLAP, which is necessary for cellular LT biosynthesis (Rouzer et al., 1990). Zileuton is a FDA-approved orally active inhibitor of 5LO and thus inhibits LTB_4_ formation. Zileuton is used to prevent difficulty in breathing, chest tightness, wheezing, and coughing due to asthma and has also been tested for its antineoplastic properties in colon, lung, and prostate cancers (Chen et al., 2004; Li et al., 2005). Inhibiting 5LO affected a lot of viral gene expression, including the lytic gene K5, which is involved in the downregulation of major histocompatibility complex class I (MHC1), intercellular adhesion molecule 1 (ICAM-1), and B7.2 (Russo et al., 1996; Neipel et al., 1997). 5LO inhibition also had a significant impact on reducing vMIP-1 and vMIP-2 gene expression as well as downregulates KSHV latent vCyclin and LANA2 gene expression (Sharma-Walia et al., 2014; Figure 4). KS lesions are prominent sites for leukocyte infiltration consisting of monocytes, lymphocytes, and mast cells (Ensoli and Sturzl, 1998; Ensoli et al., 2000; Remenyik et al., 2005). KSHV infection has been shown to shift the immune response from a TH1 to a TH2 microenvironment (Stine et al., 2000). 5LO inhibition on the other hand could reverse this by promoting TH1-related cytokines such as interferon gamma (IFNγ) and IL-2 (Figure 4). KSHV infection promotes lipogenesis (Bhatt and Damania, 2012) to aid in latency establishment (Delgado et al., 2012). Blocking 5LO reduces lipogenesis and is a target in treating obesity and its related disorders (Neels, 2013). KSHV infection promotes lipogenesis by stimulating fatty acid synthase (FASN) transcription via enhancing the levels of 5LO. Silencing 5LO reversed lipogenesis by lowering FASN promoter activity and its expression (Sharma-Walia et al., 2014). Since FASN, a key enzyme required in lipogenesis, is important in KSHV latency, study (Sharma-Walia et al., 2014) suggested that 5LO/LTB_4_ play important roles in KSHV biology and that effective inhibition of the 5LO/LTB_4_ pathway could potentially be used to control KS/PEL (Figure 4).

## Lipoxins as Anti-Inflammatory Pathways of Arachidonic Acid

**FIGURE 5:**
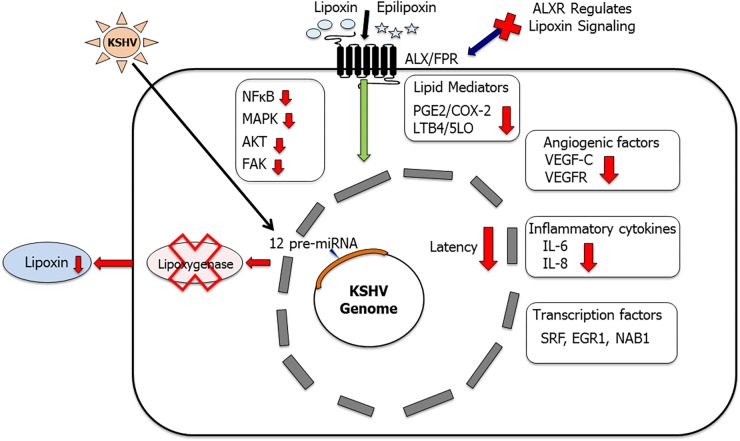
Lipoxins as anti-inflammatory lipid mediators in KSHV infection and pathogenesis: this schematic summarizes our findings (Sharma-Walia et al., 2010b; Marginean and Sharma-Walia, 2015; Chandrasekharan et al., 2016) that KSHV utilizes its miRNA cluster to target enzyme 15 lipoxygenase (15LO) to downregulate anti-inflammatory lipoxin secretion. Treating KSHV infected cells with lipoxins has been shown to provide a more resolving anti-inflammatory microenvironment (Sharma-Walia et al., 2010b; Marginean and Sharma-Walia, 2015; Chandrasekharan et al., 2016). Key inflammatory cytokines IL-6 and IL-8 were downregulated, levels of PGE_2_ and LTB_4_ were reduced, and several cellular signaling molecules such as NF-κB, AKT, FAK, and ERK levels were also lowered. Lipoxin treatment reduced angiogenic VEGF-C secretion and downregulated the phosphorylation of VEGFR (Marginean and Sharma-Walia, 2015). Lipoxin receptor ALX is a vital component of lipoxin signaling (Chandrasekharan et al., 2016). ALX silencing enhanced KSHV infection-induced NF-κB and ERK activation (Chandrasekharan et al., 2016). Lipoxin treatment also regulates KSHV infection-induced multiple transcription factors such as SRF, EGR1, and NAB1 (Marginean and Sharma-Walia, 2015).

Kaposi’s sarcoma-associated herpesvirus infection apart from triggering a lot of pro-inflammatory molecules of the arachidonic acid pathway also influences anti-inflammatory molecules such as lipoxins (Chandrasekharan et al., 2016). KS-IMM is a human Kaposi’s sarcoma tumor-derived cell line (AIDS-KS spindle cells), which was chosen as a model to study the influence of lipoxins on KSHV pathogenesis (Albini et al., 2001). KS-IMM (human Kaposi’s sarcoma tumor-derived cell line), which represents KSHV reprogrammed and transformed cells, is a spontaneously immortalized cell line obtained from a KS biopsy (Albini et al., 2001). KS-IMM cells no longer carry the KSHV genome and lack viral latency gene expression but form highly vascularized, rapidly growing tumors when injected into immunocompromised mice. In nude mice, subcutaneously injected KS-IMM cells mixed with matrigel develop palpable tumors. KS cells have been used to test the antiangiogenic, antioxidant, and anti-inflammatory potential of many clinical compounds *in vivo* (Albini et al., 1994, 2001, 2010; De Flora et al., 1996; D’Agostini et al., 1998; Cai et al., 1999; Aluigi et al., 2000; Tosetti et al., 2002; Vannini et al., 2007). It was reported that KS-IMM cells express abundant levels of enzymes COX-2 and 5LO along with their respective metabolites PGE2 and LTB_4_ (Marginean and Sharma-Walia, 2015). Similar to the previously published reports (Albini et al., 1994, 1995, 2001; Morini et al., 1999), recent study found that KS-IMM cells have a rich angiogenic and inflammatory microenvironment comprised of cytokines such as IL-6, IL-8, and VEGF-A and VEGF-C (Marginean and Sharma-Walia, 2015). Treating these cells with lipoxins decreased the levels of enzymes COX-2, 5LO, and their metabolites PGE_2_ and LTB_4_ (Marginean and Sharma-Walia, 2015; Figure 5). PGE_2_ receptors/EPs, as previously shown to influence KSHV latency, were also affected by lipoxin treatment (Marginean and Sharma-Walia, 2015). The level of EP1 receptor was reduced while EP2 and EP4 did not change on lipoxin treatment of KS-IMM cells (Marginean and Sharma-Walia, 2015). The levels of pro-inflammatory cytokines IL-6 and IL-8 secretion were markedly reduced upon lipoxin treatment of KS-IMM cells (Marginean and Sharma-Walia, 2015; Figure 5). A significant decrease in the phosphorylation status of FAK, AKT, NF-κB, and ERK1/2 upon lipoxin treatment was observed (Marginean and Sharma-Walia, 2015). These signaling pathway molecules are generally upregulated during KSHV infection. In addition to influencing signaling molecules, lipoxin treatment also altered the levels of transcription factors to control gene expression of various pro-inflammatory molecules such as interferons, and cytokines (Marginean and Sharma-Walia, 2015). Angiogenic factor VEGF-C secretion was lowered on lipoxin treatment while VEGF-A levels were not affected (Marginean and Sharma-Walia, 2015). VEGFs are primarily involved in endothelial cell tube formation, which was abrogated in cells treated with lipoxins. VEGFs interact with VEGFRs, which consist of three types VEGF-R1, -R2, and -R3 (Neufeld et al., 1996, 1999; Poltorak et al., 1997, 2000). This interaction between VEGF and its receptor promoted KSHV pathogenesis (Montaner et al., 2001; Bais et al., 2003; Zhang et al., 2005; Sivakumar et al., 2008; Dai et al., 2012). When compared to HMVEC-d cells, KS-IMM cells express higher levels of VEGFR2 (Marginean and Sharma-Walia, 2015). Lipoxin treatment of KS-IMM cells markedly reduced the activity (phosphorylation) of VEGFR2 by translocating it from lipid raft to non-lipid raft domains (Marginean and Sharma-Walia, 2015). Lipoxins exert their anti-inflammatory activity by binding to a GPCR called lipoxin A4 receptor/formyl peptidyl receptor (ALX/FPR) (Serhan et al., 1984; Maderna and Godson, 2009). The status of ALXR/FPR was examined on KS-IMM cells. When compared to HMVEC-d cells, KS-IMM cells express reduced numbers of ALXRs (Marginean and Sharma-Walia, 2015).

This study was further expanded to a more relevant model of KSHV infection using HMVEC-d cells. *De novo* KSHV infection in HMVEC-d cells shows a time of infection dependent decrease in lipoxin secretion. Similarly, KSHV positive PEL cell lines including BCBL-1, BC-3 have a reduced level of lipoxin secretion when compared to KSHV negative BJAB cells and healthy B cells (Chandrasekharan et al., 2016). This observation shows that KSHV infection downregulates lipoxin secretion. The role of lipoxin receptor in this process was then studied. Immunohistochemistry of an array of KS patient tissue sections shows that ALXR is widely present in infected cells and the levels of ALXR/FPR in KS cells are like those in healthy cells (Chandrasekharan et al., 2016). Treating latently infected HMVEC-d cells with lipoxin/epilipoxin reduced levels of signaling molecules (NF-κB, AKT, and ERK1/2) as well as pro-inflammatory enzymes and their metabolites (COX-2/PGE_2_ and 5LO/LTB_4_; Chandrasekharan et al., 2016; Figure 5). The ALXR receptor is vital for lipoxin to exert its anti-inflammatory activity. Knocking down ALXR/FPR in Osteosarcoma U2OS cells using CRISPR/CAS9 technology affected lipoxin signaling as high levels of NFKB, AKT, ERK1/2, and inflammatory proteins (COX-2, 5LO) were still persistent in lipoxin treated cells (Chandrasekharan et al., 2016). This observation of increased levels of pro-inflammatory proteins despite lipoxin/ epilipoxin treatment could be attributed to the absence of the ALX/FPR receptor.

Since both KS and PEL are associated with latency, the influence of latency genes on lipoxin secretion was studied. Lipoxin secretion was measured in HMVEC-d cells transduced with various viral latency genes such as vCyclin, vFLIP, and LANA1. No significant change in lipoxin secretion was observed when latency genes were expressed in HMVEC-d cells (Chandrasekharan et al., 2016). The latency cluster also contains a group of KSHV 12 pre-miRNAs. To evaluate the effect of KSHV miRNA on lipoxin secretion, EA.hy 926 endothelial cells were modified lentivirally to express KSHV miRNA. As a control, EA.hy cells were lentivirally transduced with GFP. On comparing the lipoxin levels in these lentivirally modified cells, the KSHV miRNA cluster was identified as a potential cause for the observed decrease in lipoxin levels in EA.hy cells carrying KSHV miRNA (Chandrasekharan et al., 2016). miRNAs function by targeting the 3′ end of mRNA for degradation. The KSHV-miRNA cluster was found to lower levels of the enzyme 15LO, which is involved in the synthesis of lipoxins (Figure 5). This study identified that KSHV utilizes lipoxin pathway for its own advantage and treating infected cells with lipoxins could be an alternative therapeutic approach (Chandrasekharan et al., 2016). Further studies are required to understand the mechanism of lipoxins action on the KSHV life cycle. Understanding the mechanism of action of lipoxins could help to develop novel therapeutic options for KSHV infection. Our studies on KSHV and lipoxins could be extended to other viral mediated pathogenesis. Lipoxins could possibly be used in conjunction with existing therapeutics to enhance a resolving environment in PEL cells.

## Conclusion and Perspectives

Studies so far have shown that KSHV alters the lipid mediators of the arachidonic acid pathway for its own advantage. Several herpesviruses induce COX-2 expression and PGE_2_ production to enhance and establish efficient infection (Shelby et al., 2005). Like KSHV, EBV has also been shown to induce COX-2 (Gandhi et al., 2017). High levels of COX-2 were reported in Hodgkin’s lymphoma patients (Al-Salam et al., 2013). This enhanced COX-2 helps in establishing angiogenesis by upregulating VEGF. COX-2 inhibition by NS 398 has been shown to decrease VEGF levels suggesting that COX-2 plays a vital role in angiogenesis (Murono et al., 2001).

With the newly identified clinical manifestations of KSHV infection, KICS, and the IRIS, controlling inflammation could be a key strategy for treating KSHV infected patients (Gasperini et al., 2012; Polizzotto et al., 2012; Ray et al., 2012; Uldrick et al., 2012). KS and IRIS patients show high morbidity (Chandrasekharan et al., 2016). KICS is expected to be an unknown cause of mortality in HIV patients. KICS patients show extremely high levels of IL-6 and IL-10 along with other inflammatory cytokines (Polizzotto et al., 2012; Ray et al., 2012). Arachidonic acid, being a key pathway in inflammation and resolution, is a potential solution to combat KSHV associated pathogenesis. Although KSHV utilizes the pro-inflammatory arm of the arachidonic acid pathway to promote inflammation but anti-inflammatory metabolites from this pathway could be beneficial to treat/resolve inflammation induced by KSHV infection. Hence, understanding how every molecule of this pathway interacts with KSHV is essential to design therapeutic strategies. The arachidonic acid pathway provides multiple ways of targeting the virus. On one hand, using inhibitors for the inflammatory metabolites/enzymes and on the other hand promoting anti-inflammatory lipid mediators can provide benefits. Further studies are required to understand the other lipid mediators of this pathway and its relation to KSHV. With limitations in the current treatment options for KS and PEL, there is an urge to develop safer, more powerful therapeutics. Although the introduction of HAART therapy significantly reduced death due to KSHV infection, and KSHV is therefore no longer a threat to human health but treating advanced cases of KS, which do not respond to any treatment, is still challenging. Additionally, understanding viral interaction with the host is essential as similar pathways are involved in other pathogenic infections. Current treatment strategies for KS and PEL involve the use of conventional chemotherapeutics such as anthracyclines, antimitotic agents, and microtubule stabilizers, and CHOP (cyclophosphamide, doxorubicin, vincristine, and prednisone) which provide no specific cure for PEL (Okada et al., 2014). Many studies describing specific anti-PEL therapies are underway, which include development of pro-apoptotic agents (proteasome inhibitor bortezomib and azidothymidine), anti-proliferative antibiotic rapamycin, anti-proliferative (PI3K/AKT, NFkB inhibitors), p53 activator nutlin-3a, antiviral compounds cidofovir and interferon-α, and KSHV latency gene blocking agents such as glycyrrhizic acid (GA), and G-quadruplex stabilizers (Hocqueloux et al., 2001; Toomey et al., 2001; An et al., 2004; Curreli et al., 2005; Klass and Offermann, 2005; Luppi et al., 2005; Halfdanarson et al., 2006; Sarek and Ojala, 2007; Sarek et al., 2007; Sin et al., 2007; Carbone et al., 2009; Bhatt et al., 2010; Madireddy et al., 2016). Antiviral drugs such as acyclovir, adefovir, foscarnet, and cidofovir are in clinical use and have shown moderate benefits in reducing viral load (Hocqueloux et al., 2001; Luppi et al., 2005; Halfdanarson et al., 2006). KSHV is commonly co-infected with HIV and effective control of the latter by HAART therapy has been shown to be effective; however, treating advanced KS remains a problem (Casper, 2008).

## Author Contributions

Both authors equally contributed to review organization, concept, interpretation of studies, and drafting the figures.

## Conflict of Interest Statement

The authors declare that the research was conducted in the absence of any commercial or financial relationships that could be construed as a potential conflict of interest.

## Abbreviations

cAMP: cyclic AMP
COX: cyclooxygenase
EGF: epidermal growth factor
FAK: focal adhesion kinase
FLAP: 5-lipoxygenase activating protein
G-CSF: granulocyte colony-stimulating factor
GM-CSF: granulocyte-macrophage colony-stimulating factor
HFF: human foreskin fibroblasts
HMVEC-d: human microvascular endothelial cells
H-PGDS: hematopoietic PGD synthase
IRIS: immune reconstitution inflammatory syndrome
KICS: KSHV inflammatory cytokine syndrome
KS: Kaposi’s sarcoma
KSHV: Kaposi’s sarcoma-associated herpesvirus
LO: lipoxygenase
LTA4H: leukotriene A4 hydrolase
LTs: leukotrienes
MCD: multicentric Castleman’s disease
MCP: macrophage chemoattractant protein
MEK: mitogen-activated protein kinase
MIP: macrophage inflammatory protein
mPGES: microsomal PGE synthase
PDGF-B: platelet-derived growth factor-B
PEL: primary effusion lymphoma
PGs: prostaglandins
PI3K: phosphoinositide-3-kinase
PKA: protein kinase A
PKC: protein kinase C
TIVE-LTC: long-term infected telomerase immortalized endothelial cells
TXS: thromboxane synthase
VEGF: vascular endothelial growth factor
VEGFR: vascular endothelial growth factor receptor
